# Characterization and Discrimination of Gram-Positive Bacteria Using Raman Spectroscopy with the Aid of Principal Component Analysis

**DOI:** 10.3390/nano7090248

**Published:** 2017-09-01

**Authors:** Alia Colniță, Nicoleta Elena Dina, Nicolae Leopold, Dan Cristian Vodnar, Diana Bogdan, Sebastian Alin Porav, Leontin David

**Affiliations:** 1 National Institute for Research and Development of Isotopic and Molecular Technologies, 67-103 Donat, 400293 Cluj-Napoca, Romania; nicoleta.dina@itim-cj.ro (N.E.D.); diana.bogdan@itim-cj.ro (D.B.); sebastian.porav@itim-cj.ro (S.A.P.); 2 Faculty of Physics, Babeş-Bolyai University, 1 Kogălniceanu, 400084 Cluj-Napoca, Romania; nicolae.leopold@phys.ubbcluj.ro (N.L.); leontin.david@phys.ubbcluj.ro (L.D.); 3 Department of Food Science, University of Agricultural Science and Veterinary Medicine, 3-5 Calea Mănăştur, 400372 Cluj-Napoca, Romania; dan.vodnar@usamvcluj.ro; 4 Faculty of Biology and Geology, Babeș-Bolyai University, 44 Republicii, 400015 Cluj-Napoca, Romania

**Keywords:** Gram-positive bacteria, *L. casei*, *L. monocytogenes*, Raman scattering, SERS, silver nanoparticles

## Abstract

Raman scattering and its particular effect, surface-enhanced Raman scattering (SERS), are whole-organism fingerprinting spectroscopic techniques that gain more and more popularity in bacterial detection. In this work, two relevant Gram-positive bacteria species, *Lactobacillus casei* (*L. casei*) and *Listeria monocytogenes* (*L. monocytogenes*) were characterized based on their Raman and SERS spectral fingerprints. The SERS spectra were used to identify the biochemical structures of the bacterial cell wall. Two synthesis methods of the SERS-active nanomaterials were used and the recorded spectra were analyzed. *L. casei* and *L. monocytogenes* were successfully discriminated by applying Principal Component Analysis (PCA) to their specific spectral data.

## 1. Introduction

In the last years, a continued growing interest was shown towards the development of new routine detection methods for microorganisms’ identification, which are fast, cost-effective, and more sensitive than the conventional ones (e.g., polymerase chain reaction [1,2,3,4,5], matrix-assisted laser desorption/ionization time-of-flight mass spectrometry [6], enzyme-linked immunosorbent assay (ELISA) technique [5,7,8,9], etc.). As an alternative to conventional methods, vibrational spectroscopy-based detection techniques, such as surface-enhanced Raman scattering (SERS) spectroscopy, gained a lot of attention and popularity in food applications [10,11], due to the minimal sample preparation and the ability to probe samples under in vivo conditions without the necessity of markers [12]. SERS technique is considered to be a fast, reliable, and highly sensitive tool for bacteria detection, down to a single cell in food and water safety controls [13,14,15,16,17,18], and involves the interaction of molecules with a metallic surface [13,19]. Among the SERS active nanomaterials, the metallic nanoparticles (colloidal suspensions) have been intensively exploited as SERS signal boosters that can be easily synthesized at room temperature [20,21], with tunable size and morphology, depending on their final application in detection [22,23,24,25,26].

The in situ approach to obtain Ag-based SERS nanomaterials was firstly reported by Efrima et al. [27], and intensively exploited by Haisch and his collaborators [13,28,29,30,31]. Practically, the in situ generated silver nanoparticles (AgNPs) ensure intimate contact with the bacterial membrane, and facilitate label-free detection at the single-cell level of microorganisms, independently of their taxonomic diversity, growth phase, physiological state, or culture conditions. Moreover, this ultrasensitive and reliable approach can be used for high-throughput monitoring of bacteria during infection treatment, providing a fast, spectral-specific response, reflecting their resistance to certain antibiotics [31,32,33]. We have chosen these promising SERS nanomaterials that were previously fully characterized by Haisch’s group in the mentioned studies (surface plasmonic resonance SPR properties, SERS performance in different liquid media, optimum excitation wavelength, optical and physical properties underlined by spectroscopic and analytical techniques, etc.) in order to demonstrate their wide potential and high efficiency as SERS detection platform.

*Lactobacillus casei* (*L. casei*) and *Listeria monocytogenes* (*L. monocytogenes*) are both Gram-positive, rod shaped bacteria; the first being a lactic acid bacterium with significant importance in life quality as probiotic, whilst the second one is the most commonly encountered spoilage microorganism of meat that causes listeriosis. Despite the fact that the cell wall of the majority of Gram-positive bacteria contains a multilayered peptidoglycan (murein) sacculus, it was shown [34] that *L. casei* cell wall lacks the teichoic acid moiety. The anionic character of the cell envelope is given by the lipoteichoic acid (LTA) [35]. Although the S-layer is a common wall component for *L. casei* species, the study of Vadillo-Rodriguez et al. [36] showed that *L. casei* ATCC 393 doesn’t possess an S-layer [37]. *L. monocytogenes* is a non-encapsulating bacterium with a cell wall made of a multilayered peptidoglycan decorated with teichoic and lipoteichoic acid [38]. The high density of the alternating sugar units of N-acetylglucosamine (GlcNAc or NAG) and *N*-acetylmuramic acid (MurNAc or NAM), and their limited conformational flexibility, give the stiffness and the rigidity characteristic of the bacteria [39]. Such molecular structure-related particularities should be reflected in the specific spectral features, and can facilitate the identification of the species.

The studies on *L. casei* are scarce [12,40,41,42], the majority focusing on the Fourier-transform infrared spectroscopy (FTIR) characterization or classification of *Lactobacillus* species, with the final aim of microorganisms’ detection and creation of library groups [43]. The few Raman studies on *L. casei* report the development of rapid and reagentless approaches for the discrimination and classification of several lactic acid bacterial strains from yogurt and kefir [12,40,42]. There are, however, no SERS studies reported so far on *L. casei*. Several studies of *L. monocytogenes* are reported in the literature, in which receptor-mediated detection methods [4,5,8,9,44,45,46,47,48], or label-free spectroscopic-based approaches [49,50] using 785 nm [14,44,45] or 532 nm [44] laser lines, have been used. Small disagreements concerning the spectral fingerprint of this virulent pathogen exist, due to the use of distinct excitation sources, growth conditions [51], sample pretreatment, SERS experimental approach (bulk or single-cell level) or signal processing [44].

Therefore, in this work, on the one hand, we discuss the biochemical origins of both Raman and SERS spectra of *L. monocytogenes* using the 632.8 nm laser line, and on the other hand, we embark upon the investigation of the probiotic *L. casei* by using Raman scattering and multivariate analysis. The aim of our study was to apply Raman and SERS spectroscopic techniques for detection and characterization of these two relevant Gram-positive bacteria species. Furthermore, by combining the spectroscopic methods with principal component analysis (PCA), the bacterial species discrimination was successfully carried out. Rapid differentiation of a probiotic from a pathogen species is essential, considering the fact that both could be present in the same human fluid or even milk sample. By carefully analyzing the unique spectral fingerprints with the aid of unsupervised chemometric tools, we propose a step-by-step guide for ultrasensitive detection and high-accuracy discrimination for future extended applications. The thorough comparison of two types of interaction between living microorganisms and AgNPs, depending on their synthesis method and interaction time, is a novel input for existent bacteria detection approaches. The different behavior of a probiotic and a culprit pathogen in the same experimental conditions, as presented in this work, contributes to identifying which characteristics from the unique SERS signature of Gram-positive species can be used for the rapid discrimination of a contaminated sample from a common probiotic.

## 2. Results and Discussion

### 2.1. UV/Visible Spectroscopy and Scanning Transmission Electron Microscopy (STEM)

**Figure 1:**
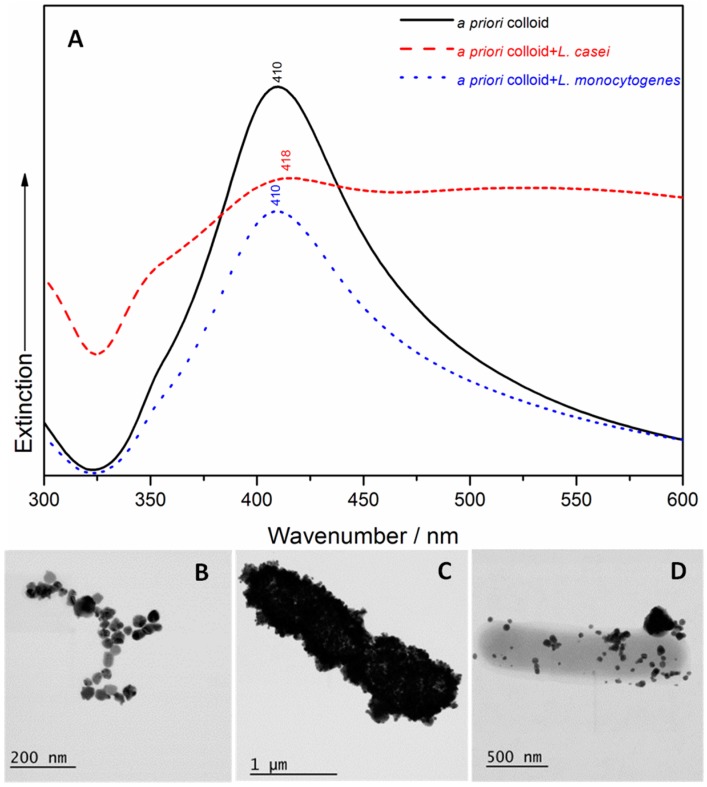
(**A**) The UV/Vis spectra of the *a priori* colloid, the *Lactobacillus casei* surface-enhanced Raman scattering (SERS) sample and the *Listeria monocytogenes* SERS sample. Scanning Transmission Electron Microscopy (STEM) images of the *a priori* colloid (**B**); the *a priori* synthesized Ag colloid coverage of the *Lactobacillus casei* (**C**) and *Listeria monocytogenes* (**D**) cell membranes.

**Figure 2:**
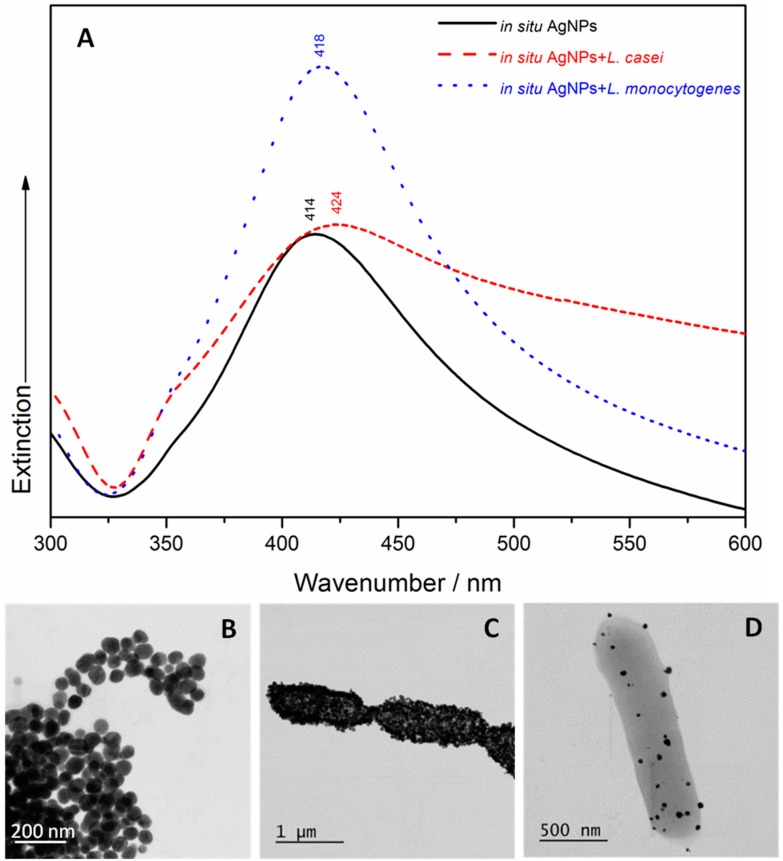
(**A**) The UV/Vis spectra of the in situ silver nanoparticles (AgNPs), the *L. casei* SERS sample and the *L. monocytogenes* SERS sample. STEM micrographs of the in situ AgNPs (**B**); the in situ AgNPs covering the *L. casei* (**C**) and *L. monocytogenes* (**D**) cell membranes.

A UV/Vis spectroscopic characterization and STEM examination of the four samples containing bacteria and the two types of SERS Ag nanomaterials were carried out. The *a priori* Ag colloid has a narrower absorption peak at 410 nm (Figure 1A) than the in situ AgNPs, with an absorption maximum that is red shifted at 414 nm (Figure 2A). These results indicate the existence of monodispersed NPs with a diameter of ≈100 nm (as resulting from the STEM image of Figure 1B) in case of the *a priori* Ag colloid, and a diameter of ≈77 nm (as shown in the STEM image of Figure 2B) in case of the in situ AgNPs. The addition of *L. casei* to the two SERS active substrates leads to a very wide broadening of the peaks’ shape, and an additional red shift to 418 nm (in case of the *a priori* Ag colloid containing sample) and to 424 nm (in case of the in situ AgNPs containing sample), respectively. In case of the *L. monocytogenes* SERS samples, a slight broadening of the UV/Vis peak was observed, with no shift (in case of the *a priori* Ag colloid containing sample) and slightly red shifted to 418 nm (in case of the in situ AgNPs containing sample). The broadening of the peak can be related to the aggregation induced by the bacteria, while the occurring red shift could indicate that the interparticle distance decreases and/or the size of the AgNPs increases. All these effects were also observed by the group of Haisch in their paper [28], in case of Gram-positive containing SERS samples, and suggested a variation in the AgNPs’ morphology, in their aggregation or assembly behavior in the bacterial/AgNPs samples [28].

### 2.2. Atomic Force Microscopy (AFM) Characterization

**Figure 3:**
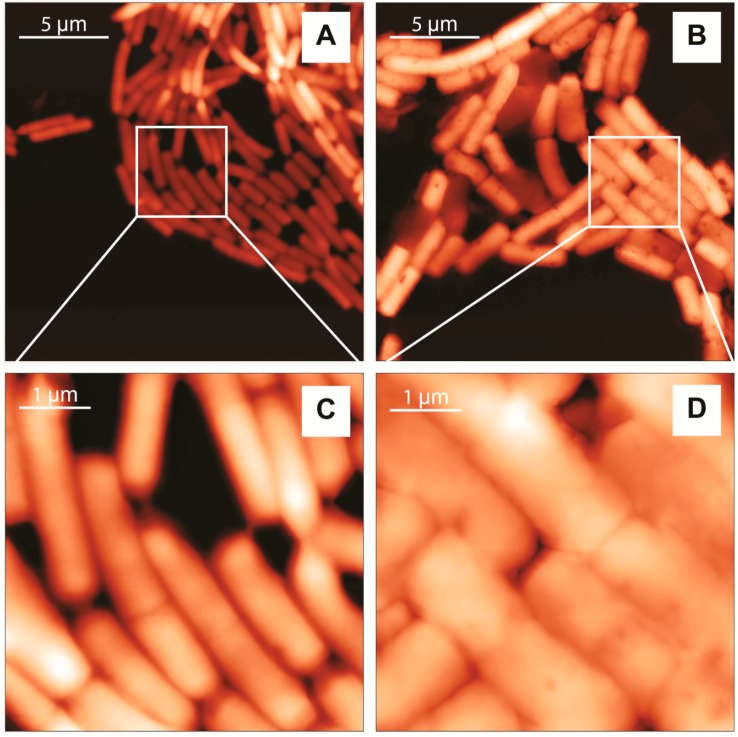
Two-dimensional topography AFM images of *L. casei* (left) and *L. monocytogenes* (right). Images were collected with a scan frequency of 0.7 Hz, 256 × 256 pixels, scan time 6 min. (**A**,**C**) and 512 × 512 pixels, scan time 13 min (**B**,**D**); The height scale is: 0–0.72 µm (**A**); 0–0.88 µm (**B**); 0–0.40 µm (**C**) and 0–0.71 µm (**D**).

The Atomic Force Microscopy (AFM) height images of *L. casei* and *L. monocytogenes* are shown in Figure 3. The cells’ shapes and surface features are similar to the AFM images of Gram-positive bacteria reported in literature [52,53]. They show a characteristic rod-shaped morphology, but the surface of the two bacterial cells was different. *L. casei* appear undamaged and with a well-defined periphery between cells, displaying a smooth and homogeneous cell surface with no evident features (Figure 3A,C). This indicates that bacteria were not deformed or damaged by the cleaning protocol and the operation conditions. On the other hand, although *L. monocytogenes* appear intact, their cell surface is rougher, with edges less defined (Figure 3B,D). These could be due to dehydration of the cells, as experiments were conducted in air on dried specimens.

AFM profile analysis also provided the bacterial cell dimensions and confirm the viability of the biological samples. The average cell size was calculated from a representative sample, and the results are shown in Table 1. Our results are similar to previously reported values [54,55].

### 2.3. Raman Measurements and Analysis

**Figure 4:**
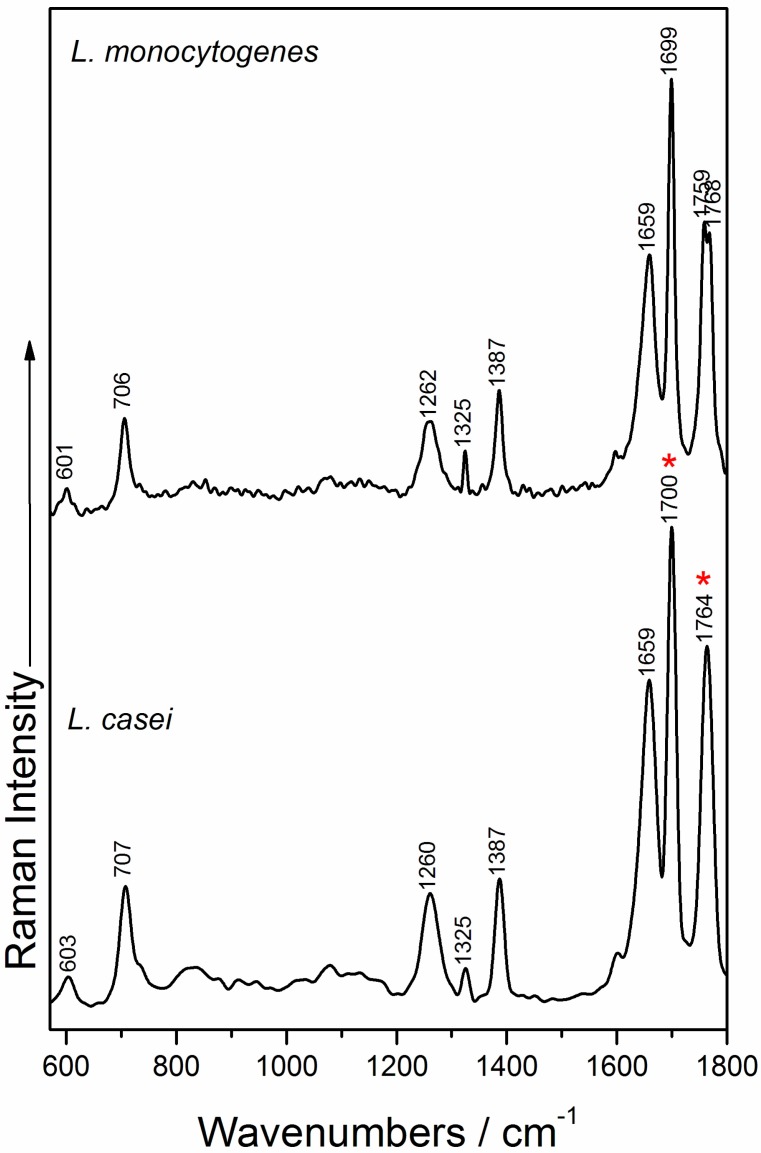
Raman spectra of *L. casei* (bottom) and *L. monocytogenes* (top).The “*” symbol indicates the Raman bands contributing to the principal component analysis (PCA) discrimination.

The information provided by typical Raman spectra of microorganisms is related to cell wall composition and cytoplasmic components. Representative Raman spectra of *L. casei* and *L. monocytogenes* are presented in Figure 4, and exhibit strongly similar spectral profiles. Therefore, in order to be able to differentiate between a probiotic and a virulent pathogen, SERS analysis is recommended, leading to an increase in the sensitivity of the measurements, due to both the electromagnetic and chemical effects produced by the presence of the silver nanoparticles, and thus, may help to differentiate the bacteria.

The Raman spectra of *L. casei* exhibit peaks located in the region 1690–1770 cm^−1^, typically assigned to C=O ester stretching group, as proposed by Maquelin et al. [56] and Lu et al. [57]. The amide I band from the proteins usually appears around 1650 cm^−1^ [12,57,58] and in our study, are registered at 1659 cm^−1^ for both species’ Raman fingerprint, while amide III band is present at 1260 cm^−1^, as also reported by Gaus et al. [42] and Mobili et al. [40]. The adenine vibrations give rise to the band observed at 707 cm^−1^, and is slightly blue shifted compared to the corresponding band acquired when using the 785 nm laser line [14,44].

In the case of the Raman spectra of *L. monocytogenes*, from the nine Raman marker bands identified by Uusitalo et al. [44] in their study on *L. innocua*, the main spectral bands that we can confirm five that are ascribed to the phenylalanine skeletal (601 cm^−1^), adenine (706 cm^−1^); amide III band (1262 cm^−1^), tyrosine and uracil nucleobases vibrations (1325 cm^−1^ band), and thymine vibrations (1387 cm^−1^ band), as also assigned by Gaus et al. [42].

All the corresponding molecules that give Raman bands in the spectra of the studied bacteria are components of the typical Gram-positive bacteria cell wall [59], as also suggested by the Raman measurements of the two bacterial culture media used in this study (Figure S1A,B). Due to the slight, or non-visible differences between the Raman spectra of the two species, statistical methods of data analysis are needed for the discrimination of the two species. In this case, the minor Raman shifting or secondary Raman bands can be analyzed and considered as Raman distinctive features for the two bacterial species. Table S1 shows the relevant Raman shifting and their assignments.

### 2.4. SERS Measurements and Analysis

**Figure 5:**
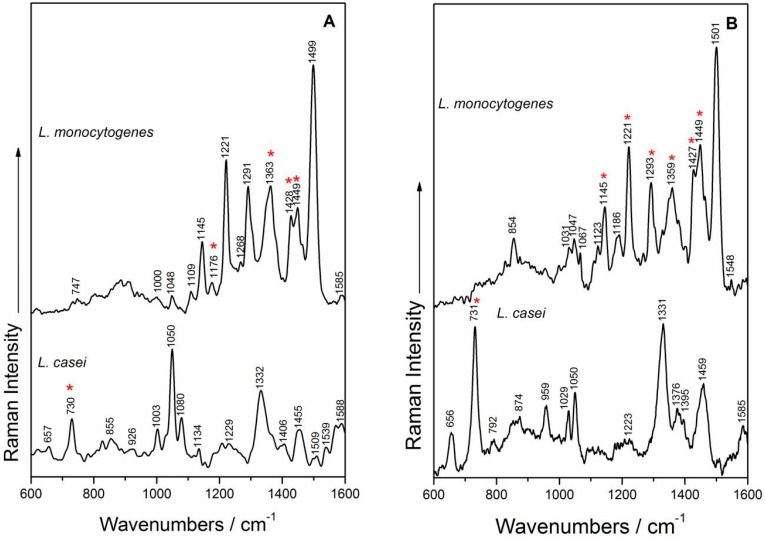
SERS spectra using *a priori* prepared Ag colloid (**A**) of *L. monocytogenes* (top) and *L. casei* (bottom) and in situ synthesized Ag nanoparticles (**B**) of *L. monocytogenes* (top) and *L. casei* (bottom). The “*” symbol indicates the SERS bands contributing to the PCA discrimination.

The SERS spectra of *L. casei* and *L. monocytogenes* obtained with *a priori* prepared colloid and the ones obtained by using in situ synthesized AgNPs are shown in Figure 5. The illustrated spectra can be analyzed from different perspectives: (i) the observation of spectral differences due to the use of two different SERS detection approaches; (ii) the comparison of the spectral fingerprint of the two species, and finally; (iii) comparing our results with previous reported results by Mircescu et al. [13] on Gram-negative bacteria using the same in situ synthesis protocol of the SERS-active substrate as in [28], in terms of feasibility of the in situ detection approach, with extended use for various strains and species of microorganisms.

*SERS spectra of L. monocytogenes*. Analyzing the SERS spectra from Figure 5, the effect of the SERS enhancement approach was found to be negligible, the spectral profiles of *L. monocytogenes* being quite independent of the AgNP synthesis method used. Moreover, it was found that the AgNPs mainly interact with the cell wall components, and the SERS spectra of *L. monocytogenes* contain all nine marker bands [44] in both SERS detection approaches (Figure 3): 1048/1047 cm^−1^ in-plane CH bending mode [14], the band at 1123/1145 cm^−1^ is the result of CN and CC stretching in carbohydrates [14,56] or in lipids [60], or even the aromatic amino acids’ deformation in proteins [61], the band at 1221 cm^−1^ is assigned to amide III [57], while the band at 1291/1293 cm^−1^ represents CH deformations in proteins [57]. The bands; at 1363/1359 cm^−1^ were assigned to the CH deformations of proteins or ν(COO^−^) symmetric deformations [58,60], whereas the 1428/1427 and 1449 cm^−1^ bands are due to CH_2_ deformations of saturated lipids [14,35]. The group of bands in the region 1400–1500 cm^−1^ is due to (CC) stretching vibrations, to (CH_2_) twist vibrations and (CH_3_) or (CH_2_) deformations, respectively fatty acids [62].

*SERS spectra of L. casei.* The two different Ag colloid syntheses influence the relative intensities of the SERS marker bands recorded for *L. casei* (Figure 5)—for example, bands ascribed to vibrations that are particularly due to purine-like molecules/adenine-containing molecules (730/731, 1050, 1332/1331 cm^−1^) [13,63,64,65,66,67,68] or CH_2_ deformations of saturated lipids (the band at 1455/1459 cm^−1^) [13,63]. This last band was also observed by Prucek et al. [69] at 1456 cm^−1^ in the SERS spectra of other Gram-positive bacteria species, and was assigned to COH vibrations of oligosaccharides. The band at 959 cm^−1^ from the in situ SERS spectrum could be assigned to the (CN) stretching vibrations, as suggested by Vohnik et al. [70] and by Kahraman et al. [61]. It is worth mentioning that the 1050 cm^−1^ band was also assigned by some authors to C–O stretching of glucose [71], as the cell wall structure of *L. casei* is made of large amounts of sugars and amino sugars.

One explanation for several spectral features that are different from published work on these species could be related to the low possibility of the herein synthesized AgNPs to reach inside the cell, in comparison to other in situ results on bacteria [27]. On the other hand, the in situ SERS results show several resemblances, but also some discrepancies, in comparison with the SERS spectra obtained by Mircescu et al. [13]. The SERS spectra of *E. coli* show multiple bands associated to the phospholipid bilayer outside of the peptidoglycan layer, as this membrane is thought to be in close proximity to the SERS substrate. The herein shown spectra contain peaks arising from the AgNPs’ interaction with the peptidoglycan layer, the lipoteichoic acids or the glucose, as the cell wall of *L. casei* was demonstrated to be made of ≈43% glucose [34].

Overall, the accuracy of the obtained SERS results is sustained by their reproducibility. Five consecutive accumulations for each bacterial sample are presented in Figure S2, and all the marker bands are present in their spectral profiles.

### 2.5. Principal Component Analysis of L. casei and L. monocytogenes Spectra

The grouping of the two Gram-positive bacteria species, and the spectral differences between them were evaluated in the principal component (PC) space by using PCA on the spectral data. So far, the discrimination of *Listeria* species was assessed by only taking into account the 600–800 cm^−1^ region of the SERS spectra [44]. In this work, we are reporting a full Raman or SERS spectra PCA analysis, without excluding specific spectral information. Satisfying accuracies for classifying *Listeria* species based only on their extended Raman spectra (400–1800 cm^−1^) was shown by Wang et al. [72].

Thus, 10 Raman spectra of *L. casei* and 23 Raman spectra of *L. monocytogenes*, within the 570–1800 cm^−1^ spectral range, were used for the PCA discrimination. In the case of the *a priori* SERS approach spectra, the PCA was applied on 145 SERS spectra of *L. casei* and 62 SERS spectra of *L. monocytogenes*, within the 600–1600 cm^−1^spectral range. Finally, using in situ synthesized AgNPs, 83 SERS spectra of *L. casei* and 11 SERS spectra of *L. monocytogenes*, within the 600–1600 cm^−1^ spectral range, were used for PCA analysis.

**Figure 6:**
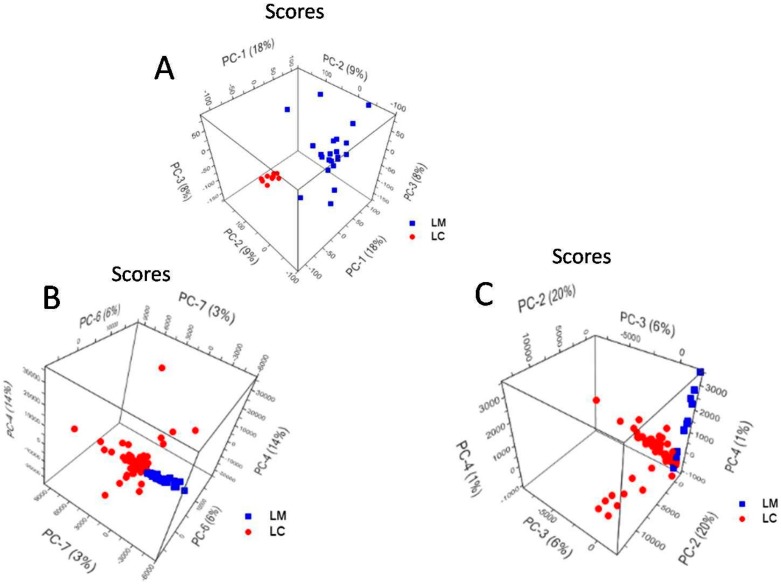
PCA scores plot of (**A**) Raman spectra; (**B**) SERS spectra using *a priori* prepared Ag colloid and (**C**) SERS spectra obtained using in situ prepared Ag colloid, showing the grouping of *L. monocytogenes* (LM) and *L. casei* (LC).

Figure 6A depicts the PCA scores plot of PC-1 vs. PC-2 and PC-3 obtained for the Raman spectra of *L. casei* and *L. monocytogenes,* explaining 35% of the total variance. The two Gram-positive bacteria are fairly separated along the PC-1 axis, which explains the 18% of the total variance in the data set. The PC-1 loadings values are plotted in Figure S3 and represent the spectral differences among the bacterial species. The highest variance values correspond to the Raman bands, assigned to C=O ester stretching group [56], and located at 1764 cm^−1^ in the *L. casei* spectrum, whereas in the *L. monocytogenes* spectrum, splits into two bands at 1759 and 1768 cm^−1^. The differentiation is also influenced by the band at 1700 cm^−1^ in the *L. casei* spectrum, which appears slightly shifted at 1699 cm^−1^ in the *L. monocytogenes* spectrum (Figure 4), and also arises from the C=O ester stretching vibrations [56].

The PCA scores plot of PC-4 vs. PC-6 and PC-7 conducted over the *a priori* SERS approach spectra (Figure 6B) show the separation between *L. casei* and *L. monocytogenes,* explaining 23% of the total variance of the data set. According to PC-4—Figure S4a, PC-6—Figure S4b and PC-7—Figure S4c loadings plots, one SERS band that participates inthe discrimination of the two bacteria is the adenine band located at 730 cm^−1^ in the *a priori* SERS spectrum of *L. casei.* The discrimination is based also on the AgNPs’ interaction with the *L. monocytogenes* cell wall components, with bands at 1363 cm^−1^, ascribed to CH deformations of proteins or ν(COO^−^) symmetric deformations [58,60], and at 1428 and 1449 cm^−1^ bands, due to CH_2_ deformations of saturated lipids [35] (PC-4—Figure S4a and PC-7—Figure S4c, respectively). PC-6 takes into account the band at 1176 cm^−1^, ascribed to phenylalanine, which appears only in the SERS spectra of *L. monocytogenes* as a distinct spectral feature relevant in the discrimination process.

The PCA scores plot of PC-2 vs. PC-3, and PC-4 conducted over the in situ SERS approach spectra, is presented in Figure 6C, and explains 27% of the total variance.

The separation between *L. casei* and *L. monocytogenes* is made along PC-2 and PC-3 axes, and explains 20% and 6%, respectively, of the total variance of the data set. According to the PC loading plots (Figure S5a–c), the spectral differences that contribute to the discrimination between the species are related to the 731 cm^−1^ adenine vibration band and the SERS bands assigned to CC, CO, COH deformations in carbohydrates [36] or C–C stretching in lipids [60] (the band at 1145 cm^−1^ from the *L. monocytogenes* spectrum), to amide III [57] (the band at 1221 cm^−1^), which is absent in the *L. casei* in situ SERS spectrum, to CH deformations in proteins [57] (the band at 1293, 1359 cm^−1^) and to CH_2_ deformations of saturated lipids [35] (the 1427 and 1449 cm^−1^ bands).

## 3. Materials and Methods 

### 3.1. Bacteria Cultivation

*L. casei ATCC 393* was purchased in lyophilized form (Microbiologics, St. Cloud, MN, USA) and grown in de Man, Rogosa, Sharpe (MRS) broth (Merck, Darmstadt, Germany). Routinely, the inoculum cultures were obtained from freeze-dried cells suspended in 5 mL MRS, incubated under aerobic conditions at 37 °C (24 h) and then sub-cultured into 95 mL MRS and incubated in the same conditions [73]. The inoculum cultures were grown for 24 h in a 200 mL Erlenmeyer flask containing 100 mL MRS, and mixed on a rotary shaker (Heidolph Unimax 1010, Schwabach, Germany) at 37 °C.

*L. monocytogenes ATCC 19115* was maintained on Oxford agar (Sifin, Berlin, Germany) plates at 4 °C. A single colony of *L. monocytogenes* was inoculated into a tube of tryptic soy broth plus 0.7% yeast extract (TSBYE) (Difco Laboratories) and incubated at 35 °C for 24 h.

The cell growth was monitored by UVspectrometry (Nanodrop ND-1000 Spectrophotometer, Nanodrop Technologies, Wilmington, DE, USA) by measuring the media optical density at 600 nm. In the case of *L. casei*, the concentration of the bacterial solution was found to be 10^14^ CFU/mL (O.D. = 0.95), while the concentration of *L. monocytogenes* solution was determined to be 10^11^ CFU/mL (O.D. = 0.7). *L. casei* and *L. monocytogenes* cultures were in stationary phases before harvesting the bacterial cells for analysis.

### 3.2. A Priori Colloid Synthesis and In Situ NPs Synthesis

Spherical silver nanoparticles (AgNPs) were synthesized using the procedure proposed by Leopold and Lendl [20] (the so-called *a priori* SERS substrate; the term “*a priori*” will be used throughout the text to describe this type of AgNP, and the samples containing the NPs prepared using this receipt). Silver nitrate (0.017 g) was dissolved in 90 mL double distilled water. In a separate recipient, 0.017 g of hydroxylamine hydrochloride was solved in 10 mL double distilled water, followed by the addition of 1.2 mL sodium hydroxide solution (1%). The hydroxylamine/sodium hydroxide solution was then added rapidly to the silver nitrate solution under vigorous stirring. After a few seconds, a grey-brown colloidal solution resulted, and it was further stirred for 10 min. The pH value of the silver colloid, measured immediately after preparation, was found to be 10. No specific Raman bands from the colloidal suspension were recorded, as shown in Figure S6.

The in situ NPs were obtained using the protocol described by Zhou et al. [28] and Dina et al. [30], and follows a modified procedure of Leopold and Lendl [20] (the so-called in situ SERS substrate; the term in situ will be used throughout the text to describe this type of AgNP and the samples containing the NPs prepared using this receipt). The silver nitrate solution (component A) was prepared by solving 0.017 g silver nitrate in 10 mL double distilled water, while a second component (B) resulted from the addition of 1.2 mL sodium hydroxide (1%) to a solution containing 0.012 g of hydroxylamine hydrochloride and 90 mL double distilled water. An initial test was successfully done by adding 900 μL of B to 100 μL of A, which resulted in a dark, grey-brownish suspension, an indicator of the AgNPs’ in situ synthesis.

### 3.3. Raman and SERS Measurement Details

#### 3.3.1. Instrumentation

The confocal Raman and SERS high spatial resolution (<1 μm lateral and <2 μm in depth) spectra on bacteria were recorded by using a conventional Renishaw inVia Raman Microscope(Renishaw PLC, New Mills Wotton-under-Edge, Gloucestershire, UK) equipped with a HeNe laser (632.8 nm) with a total laser power of 50 mW and a grating of 1800 L/mm. In order to avoid sample photodegradation, a neutral density filter was used, and 1% of the laser power reached the sample (0.5 mW laser power and ≈70 μW effective laser power on the sample). The instrument was wavelength calibrated using a silicon wafer.

#### 3.3.2. Sample Preparation

For the Raman, SERS, and AFM measurements, 2 mL of bacteria culture were centrifuged (6000 rpm, 10 min) and the culture medium was removed. The cell pellet was washed three times with 2 mL of MilliQ water (18 MΩ·cm^−1^ resistivity), and centrifuged in the aforementioned conditions. The harvested pellet of *L. casei* was resuspended in 1 mL MilliQ water (the final bacterial concentration was 2 × 10^14^ CFU/mL), while the harvested pellet of *L. monocytogenes* was resuspended in 250 μL MilliQ water for all the measurements (the final bacterial concentration was 8 × 10^11^ CFU/mL).

The *a priori* SERS suspensions of both *L. casei* and *L. monocytogenes* were prepared by mixing 500 μL *a priori* prepared Ag colloid with 5 μL bacteria, with a final bacterial concentration of 1.98 × 10^12^ CFU/mL and 0.8 × 10^10^ CFU/mL, respectively. The in situ SERS samples were prepared in two steps: firstly, 5 μL of washed bacteria were added to 100 μL of silver nitrate solution, followed by vigorous stirring and an interaction time of 5 min; secondly, 900 μL of hydroxylamine hydrochloride and sodium hydroxide solution was pipetted into the prepared mixture, and the mixture was again vigorously stirred. The resulting suspension had a similar color as the initial test, indicating the AgNPs’ synthesis in the presence of the bacteria pellet. The optical properties of the in situ synthesizedNPs in the presence of the bacteria were reproduced as reported in earlier work [29].

The Raman and SERS actual measurements on the obtained samples were assessed at approximately one and a half hours after the bacteria harvesting, in ambient conditions of temperature and humidity.

#### 3.3.3. Spectra Acquisition Details

All the spectroscopic experiments were carried out onto MgF_2_ slides, by drying 5 μL of washed bacteria/SERS suspension and focusing under the 50× and 100× objectives. The results presented in this work were acquired on bacterial clusters containing from one bacterium to a few dozen bacteria, in correlation with the laser spot dimension.

Point-to-point reproducibility confirmation was performed by acquisition of at least 10 spectra from different points spotted on the MgF_2_ slide (N_Raman_ = 9, N_SERS_ = 23) and all spectra were centralized onto three databases, specific for each investigation (Raman, SERS *a priori* and SERS in situ, respectively). Batch-to-batch reproducibility was accomplished by acquiring SERS spectra from more than 20 batches of independent stock inoculations of the same samples, in different days, in order to monitor day to day reproducibility of the results.

The Raman spectra were collected as extended scan in the 570–1800 cm^−1^ wavenumber range, and each Raman spectrum is the result of 10 accumulations of 10 s integration time/accumulation. To remove the background noise, a 5 points FFT smoothing correction was applied on the measured Raman spectra.

The SERS spectra were collected as static spectra in the 600–1600 cm^−1^ wavenumber range. Each SERS spectrum is a result of 3 accumulations of 5 s integration time/accumulation. Particularly, for the SERS spectra, a baseline correction in 10 points and a FFT smoothing correction in 5 points for noise reduction were applied.

The WiRE™ software package (Renishaw PLC, New Mills Wotton-under-Edge, Gloucestershire, UK) was used for instrument control and data capture. To increase signal to noise ratio, fivespectra for each sample were collected in the same experimental conditions, and the average spectrum is depicted. The final step of data manipulation consisted in the normalization to the peak with the highest intensity, whose value was set to 1.

### 3.4. UV Characterization

The UV/Vis measurements were done using a JASCO V-550 UV/Vis (JASCO International Co. Ltd, Sapporo, Japan) spectrophotometer in the wavenumber range 300–600 nm, using a tungsten lamp and a 0.1 nm resolution, operating in the absorption mode.

### 3.5. STEM Measurements

A drop of suspension of each sample was deposited and dried on a copper grid coated by a thin carbon film prior to the electron microscopy analysis. The analysis was carried out using a Hitachi HD-2700 (Hitachi, Tokyo, Japan) scanning transmission electron microscope equipped with a cold field emission gun, working at an acceleration voltage of 200 kV and designed for high-resolution (HRTEM) imaging with a resolution of 0.144 nm. Images where recorded using Digital Micrograph software from Gatan (Pleasanton, CA, USA).

### 3.6. Computational Details

The software Unscrambler^®^ X Version 10.4 (Camo Software AS., Oslo, Norway) was used to perform the PCA analysis. The pre-processing of the SERS spectral database consisted of using multiplicative scattering correction (MSC), baseline correction and Savitzky-Golay smoothing prior to the PCA analysis.

## 4. Conclusions

Two Gram-positive bacteria species were successfully characterized and discriminated using Raman and SERS spectroscopic methods, with the aid of PCA unbiased analysis. The Raman spectra of *L. casei* and *L. monocytogenes* are very similar in terms of band position and their intensity. The spectra are composed of bands arising mainly from adenine and purine-like molecules, and proteins (amide bands), but also from the unsaturated lipids from the peptidoglycan layer. The existing differences between *L. casei* and *L. monocytogenes* spectra were fairly considered in the discrimination process only when applying PCA on Raman spectra.

In case of the SERS detection, two approaches for obtaining SERS-active materials were employed. All the SERS spectra contain the expected bacterial marker bands, independently of the used AgNP synthesis method. Major relative intensity differences were observed in the SERS spectra of *L. casei*, when using *a priori* and in situ SERS colloid, and suggest different interacting mechanisms with the SERS active nanomaterials, in the case of this type of bacteria. The SERS spectra of *L. monocytogenes* are very similar, independent of the SERS substrate and composed of bands arising from the cell wall components, thus suggesting the dominant interaction of AgNPs mainly with the peptidoglycan layer and the lipid components. The two different SERS substrate synthesis protocols provided similar and reliable information that enabled bacterial differentiation using PCA analysis by explaining around 30% of the total variance.

By comprehending and then properly selecting the SERS-based detection approach when dealing with relevant microorganisms, the chances to actually employ SERS as an alternative in future clinical routine analysis are enhanced. In this case, the in situ approach looks more promising, and is currently under testing for real-life samples in our group.

## Figures and Tables

**Table 1:** Average cell size for *L. casei* and *L. monocytogenes* as obtained from AFM topographic data.

Bacteria Species	*L. casei*	*L. monocytogenes*
	Dimension (µm)	
Length	2.01 ± 0.42	2.14 ± 0.33
Width	0.72 ± 0.11	1.12 ± 0.07

## Supplementary Materials

The following are available online at http://www.mdpi.com/2079-4991/7/9/248/s1, Figure S1: Raman spectra registered for the bacterial culture media used: MRS (A) and TSBYE (B); Figure S2: Representative SERS spectra showing the reproducibility for each bacterial species—*L. casei* (right) and *L. monocytogenes* (left), on clustered bacteria, in the *a priori* (A) and in situ (B) conditions, respectively. Inset showing the laser spotlight on a bacterial cluster; Figure S3: The PC-1 loadings plot corresponding to the PCA analysis on the Raman recorded spectra—Figure 6A; Figure S4: (a) The PC-4 loadings plot corresponding to the PCA analysis on the SERS *a priori* recorded spectra—Figure 6B, (b) The PC-6 loadings plot corresponding to the PCA analysis on the SERS *a priori* recorded spectra—Figure 6B, (c) The PC-7 loadings plot corresponding to the PCA analysis on the SERS *a priori* recorded spectra—Figure 6B; Figure S5: (a) The PC-2 loadings plot corresponding to the PCA analysis on the SERS in situ recorded spectra—Figure 6C, (b) The PC-3 loadings plot corresponding to the PCA analysis on the SERS in situ recorded spectra—Figure 6C, (c) The PC-4 loadings plot corresponding to the PCA analysis on the SERS in situ recorded spectra—Figure 6C; Figure S6: The 632.8 nm Raman spectrum of the Ag colloid, Table S1: Raman and SERS bands detected for *L. casei/L. monocytogenes*.
