# Supplementary material for: Characterization and Discrimination of Gram-Positive Bacteria Using Raman Spectroscopy with the Aid of Principal Component Analysis
Source: Nanomaterials (Basel). 2017 Sep 1;7(9):248. doi: 10.3390/nano7090248 (PMC5618359; doi:10.3390/nano7090248)
Supplement: Supplementary file 1 [file nanomaterials-07-00248-s001.pdf]

Supplementary Materials: Characterization and Discrimination of Gram—Positive Bacteria Using Raman Spectroscopy with the Aid of Principal Component Analysis

Alia Colniță, Nicoleta Elena Dina, Nicolae Leopold, Dan Vodnar, Diana Bogdan, Sebastian Porav and Leontin David

Table S1. Raman and SERS bands detected for *L. casei*/*L. monocytogenes.*

Raman Bands/cm^{-1}	SERS Bands/cm^{-1}		Tentative Assignments	Reference
	a Priori Colloid	In Situ Colloid		
603/601			phenylalanine (skeletal)	[1]
	657/-	656/-	β-D-glucose	[2]
			guanine, tyrosine	[3]
707/706	730/747	731/-	ν (adenine), purine-like molecules	[1,4–10]
		854/854	aminoacids	[2]
		959/-	ν (CN)	[6,11]
	1003/1000		phenylalanine (the symmetric ring breathing mode)	[10]
		1029/1031	β-D-glucose	[2]
			ip(CH)	[10]
	1050/1048	1050/1047	ν (adenine)	[4–9]
			ν (CO) glucose	[12]
	1080/-		amino sugars	[2]
	-/1109		ν (DNA, RNA)	[13]
			ν (CN, CC) carbohydrates	[5,14]
	1134/1145	-/1123, 1145	ν (CN, CC) lipids	[15]
			aromatic amino acids in proteins	[11]
	-/1176	-/1186	phenylalanine	[2]
1260/1262	1229/1221	1223/1221	amide III	[1,13,16,17]
	-/1291	-/1293	δ (CH) proteins<br>ν_{sym} (COO^-)	[13,15,18]
1325/1325	1332/-	1331/-	ν (tyrosine, uracil)	[16]
			ν (adenine)	[4-9,12]
	-/1363	1376/1359	δ (CH) proteins<br>ν_{sym} (COO^-)	[2,15]
1387/1387			ν (thymine)	[16]
	1406/-<br>-/1428	-/1427	δ (CH_2) saturated lipids	[5,6,10,15]
	1455/1449	1459/1449	ν (CC), τ (CH2), δ (CH_3, CH_2) fatty acids	[19]
	1509/1499	-/1501	ν (COH) oligosaccharides	[20]
	1539/-	-/1548	adenine-containing molecules	[2]
	1588/1585	-/1585	proteins, aminoacids ν_{asym} (COO^-), nucleic acids	[8,10]
			guanine	[12]
1659/1659			amide I band, unsaturated lipids	[2,13,21]
1700/1699<br>1764/1759<br>1764/1768			ν (C=O) ester	[13,14]

Symbols: ν—stretching; τ—twisting; δ—deformation; ip—in plane; sym—symmetric; asym—asymmetric.*

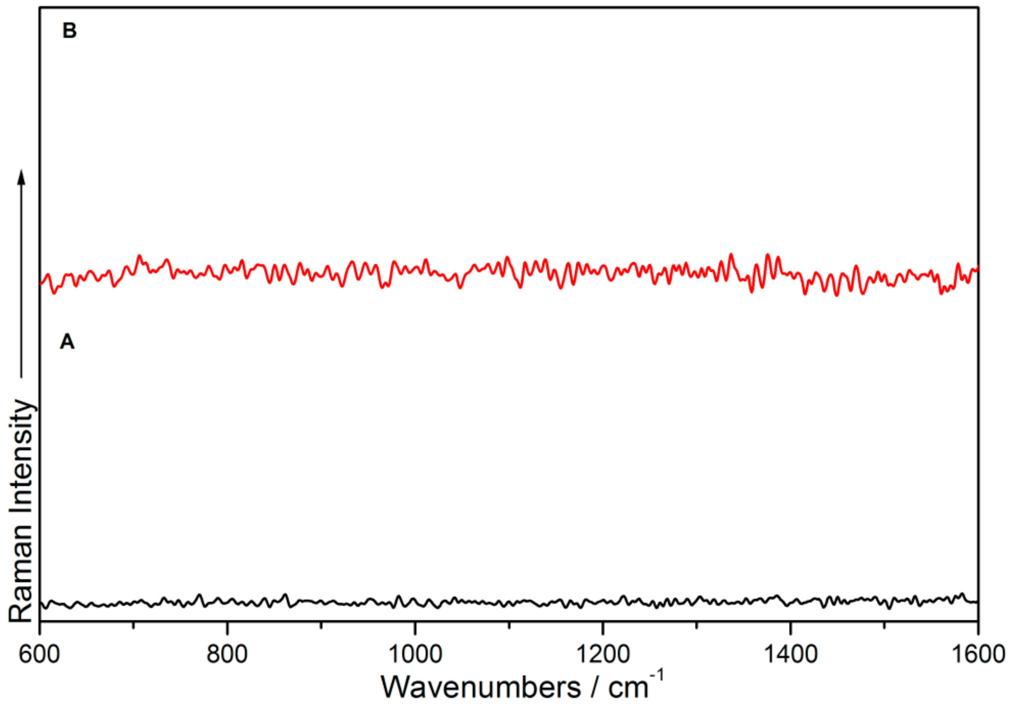

Figure S1. Raman spectra registered for the bacterial culture media used: MRS (**A**) and TSBYE (**B**).

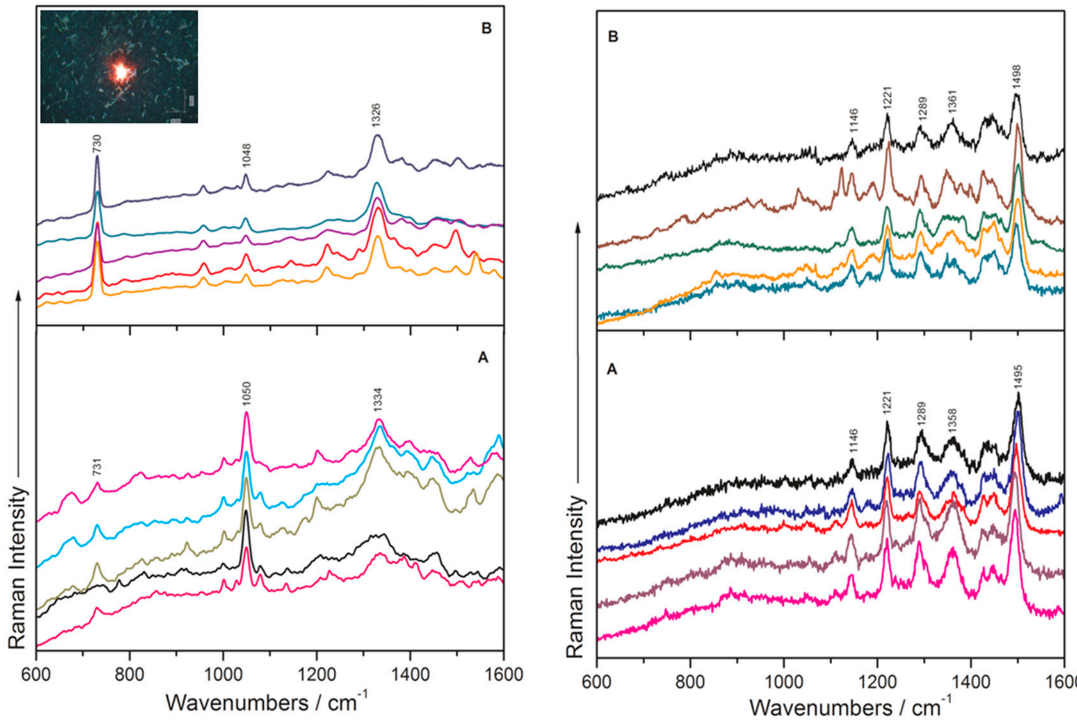

Figure S2. Representative SERS spectra showing the reproducibility for each bacterial species—*L. casei* (right) and *L. monocytogenes* (left), on clustered bacteria, in the *a priori* (**A**) and *in situ* (**B**) conditions, respectively. Inset showing the laser spotlight on a bacterial cluster.

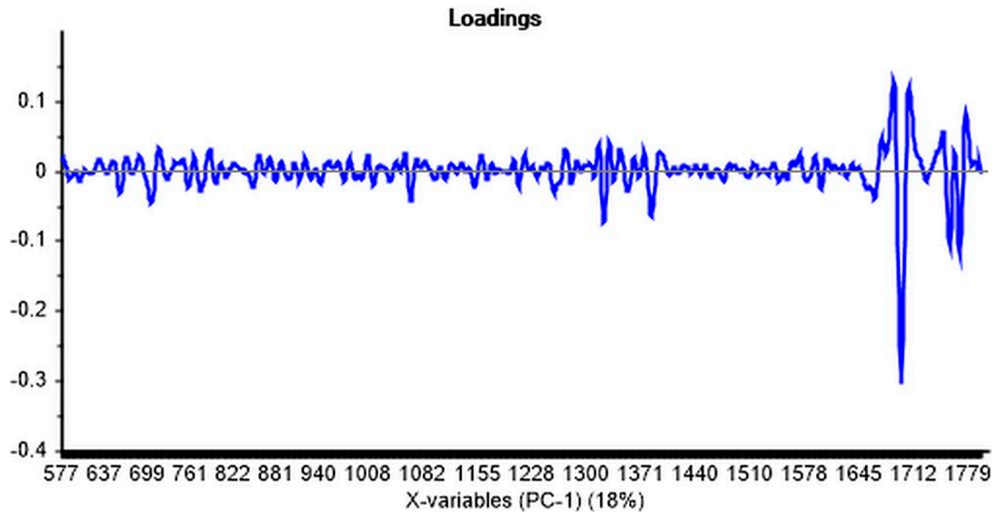

Figure S3. The PC-1 loadings plot corresponding to the PCA analysis on the Raman recorded spectra—Figure 6A.

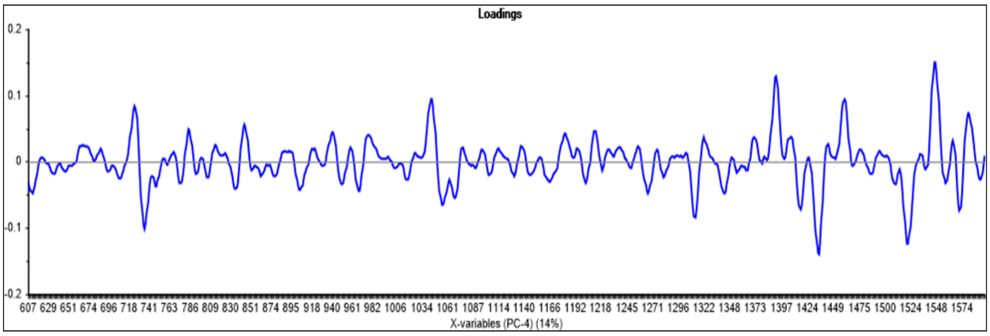

(**a**)

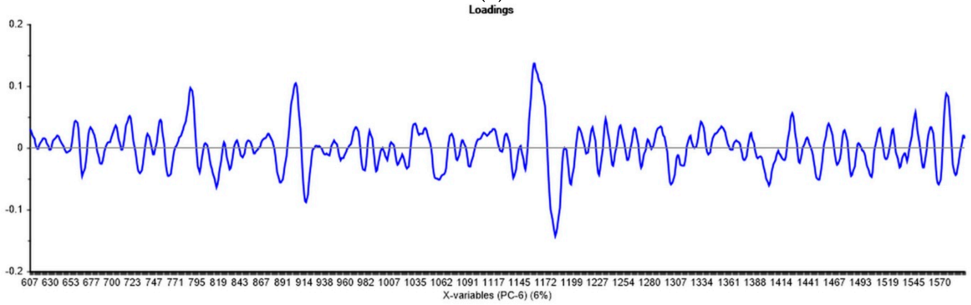

(**b**)

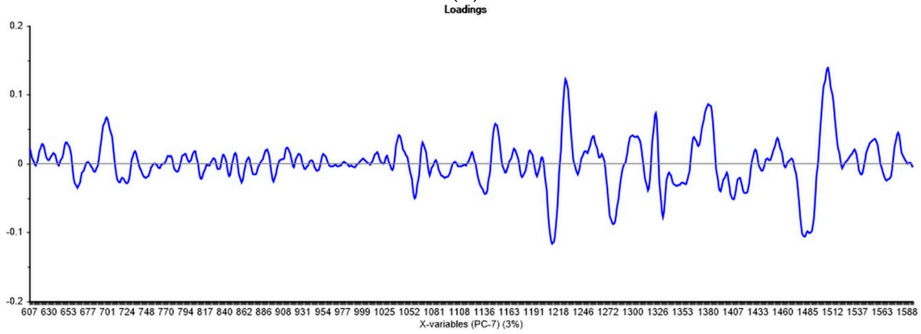

(**c**)

Figure S4. (**a**) The PC-4 loadings plot corresponding to the PCA analysis on the SERS *a priori* recorded spectra—Figure 6B; (**b**) The PC-6 loadings plot corresponding to the PCA analysis on the SERS *a priori* recorded spectra—Figure 6B; (**c**) The PC-7 loadings plot corresponding to the PCA analysis on the SERS *a priori* recorded spectra—Figure 6B.

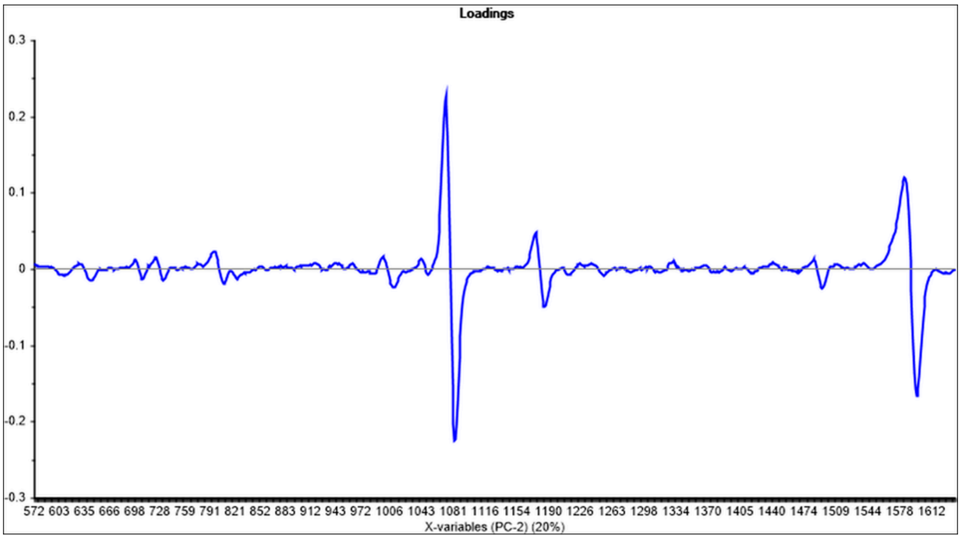
Loadings
0.3
0.2
0.1
0
-0.1
-0.2
-0.3
572 603 635 666 698 728 759 791 821 852 883 912 943 972 1006 1043 1081 1116 1154 1190 1226 1263 1298 1334 1370 1405 1440 1474 1509 1544 1578 1612
X-variables (PC-2) (20%)

(**a**)

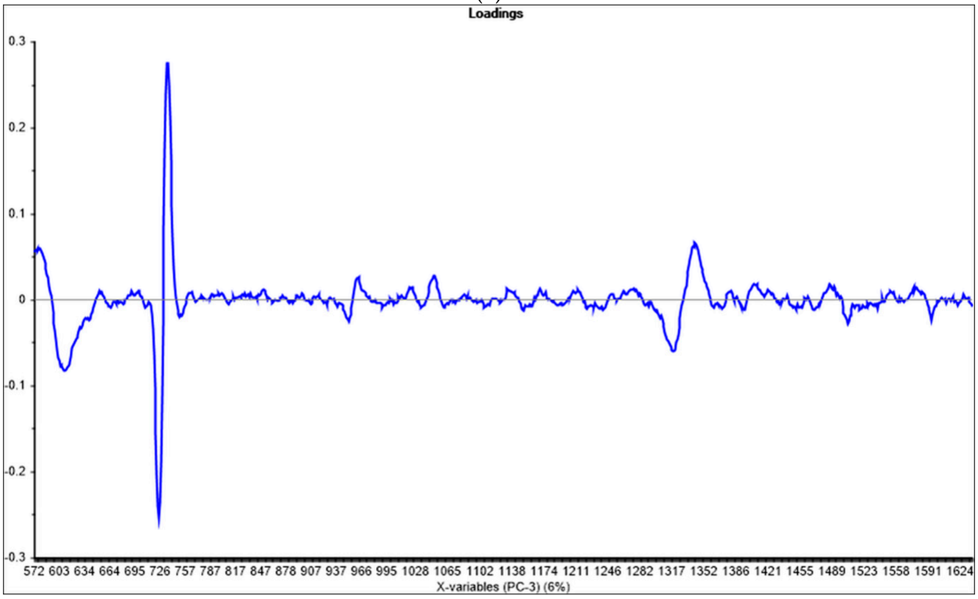
Loadings
0.3
0.2
0.1
0
-0.1
-0.2
-0.3
572 603 634 664 695 726 757 787 817 847 878 907 937 966 995 1028 1065 1102 1138 1174 1211 1246 1282 1317 1352 1386 1421 1455 1489 1523 1558 1591 1624
X-variables (PC-3) (6%)

(**b**)

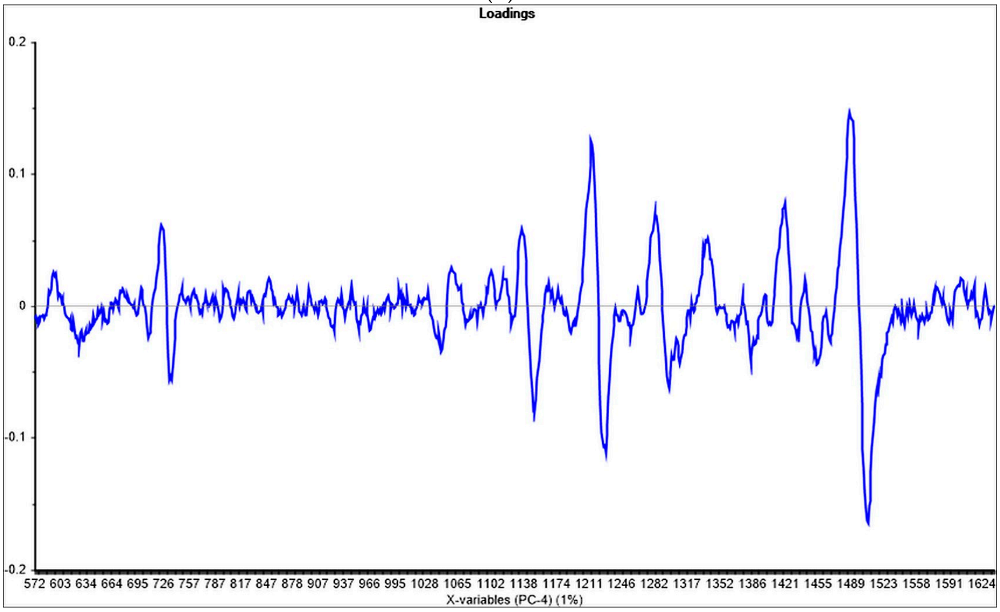
Loadings
0.2
0.1
0
-0.1
-0.2
572 603 634 664 695 726 757 787 817 847 878 907 937 966 995 1028 1065 1102 1138 1174 1211 1246 1282 1317 1352 1386 1421 1455 1489 1523 1558 1591 1624
X-variables (PC-4) (1%)

(**c**)

Figure S5. (**a**) The PC-2 loadings plot corresponding to the PCA analysis on the SERS *in situ* recorded spectra—Figure 6C; (**b**). The PC-3 loadings plot corresponding to the PCA analysis on the SERS *in situ* recorded spectra—Figure 6C; (**c**) The PC-4 loadings plot corresponding to the PCA analysis on the SERS *in situ* recorded spectra—Figure 6C.

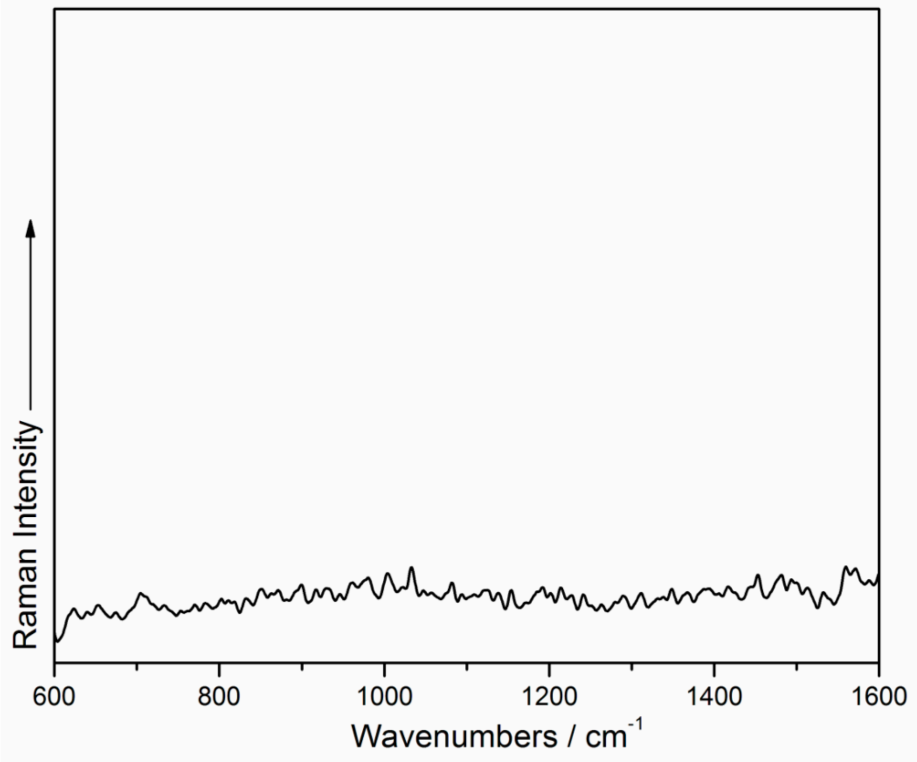

Figure S6. The 632.8 nm Raman spectrum of the Ag colloid.

References

1. Uusitalo, S.; Kogler, M.; Valimaa, A.L.; Popov, A.; Ryabchikov, Y.; Kontturi, V.; Siitonen, S.; Petaja, J.; Virtanen, T.; Laitinen, R.; et al. Detection of *Listeria innocua* on roll-to-roll produced SERS substrates with gold nanoparticles. *RSC Adv.* **2016**, *6*, 62981–62989.
2. De Gelder, J.; De Gussem, K.; Vandenabeele, P.; Vancanneyt, M.; De Vos, P.; Moens, L. Methods for extracting biochemical information from bacterial Raman spectra: focus on a group of structurally similar biomolecules-fatty acids. *Anal. Chim. Acta* **2007**, *603*, 167–175.
3. Zhou, H.; Yang, D.; Ivleva, N.P.; Mircescu, N.E.; Niessner, R.; Haisch, C. SERS detection of bacteria in water by *in situ* coating with Ag nanoparticles. *Anal. Chem.* **2014**, *86*, 1525–1533.
4. Premasiri, W.R.; Lee, J.C.; Sauer-Budge, A.; Théberge, R.; Costello, C.E.; Ziegler, L.D. The biochemical origins of the surface-enhanced Raman spectra of bacteria: A metabolomics profiling by SERS. *Anal. Bioanal. Chem.* **2016**, *408*, 4631–4647.
5. Mircescu, N.E.; Zhou, H.; Leopold, N.; Chis, V.; Ivleva, N.P.; Niessner, R.; Wieser, A.; Haisch, C. Towards a receptor-free immobilization and SERS detection of urinary tract infections causative pathogens. *Anal. Bioanal. Chem.* **2014**, *406*, 3051–3058.
6. Walter, A.; Marz, A.; Schumacher, W.; Rosch, P.; Popp, J. Towards a fast, high specific and reliable discrimination of bacteria on strain level by means of SERS in a microfluidic device. *Lab Chip* **2011**, *11*, 1013–1021.
7. Kahraman, M.; Keseroglu, K.; Culha, M. On sample preparation for surface-enhanced Raman scattering (SERS) of bacteria and the source of spectral features of the spectra. *Appl. Spectrosc.* **2011**, *65*, 500–506.
8. Ivleva, N.P.; Wagner, M.; Szkola, A.; Horn, H.; Niessner, R.; Haisch, C. Label-free in situ SERS imaging of biofilms. *J. Phys. Chem. B* **2010**, *114*, 10184–10194.
9. Zeiri, L.; Bronk, B.V.; Shabtai, Y.; Eichler, J.; Efrima, S. Surface-enhanced Raman spectroscopy as a tool for probing specific biochemical components in bacteria. *Appl. Spectrosc.* **2004**, *58*, 33–40.

10. Beuchat, L.R.; Doyle, M.P.; Montville, T.J. *Food Microbiology: Fundamentals and Frontiers*. 2nd ed.; ASM Press: Washington, DC, USA, 2001.
11. Kahraman, M.; Zamaleeva, A.I.; Fakhrullin, R.F.; Culha, M. Layer-by-layer coating of bacteria with noble metal nanoparticles for surface-enhanced Raman scattering. *Anal. Bioanal. Chem.* **2009**, *395*, 2559–2567.
12. Sivakesava, S.; Irudayaraj, J.; Ali, D. Simultaneous determination of multiple components in lactic acid fermentation using FT-MIR, NIR, and FT-Raman spectroscopic techniques. *Process Biochem.* **2001**, *37*, 371–378.
13. Lu, X.; Al-Qadiri, H.; Lin, M.; Rasco, B. Application of Mid-infrared and Raman Spectroscopy to the Study of Bacteria. *Food Bioprocess. Technol.* **2011**, *4*, 919–935.
14. Maquelin, K.; Kirschner, C.; Choo-Smith, L.P.; van den Braak, N.; Endtz, H.P.; Naumann, D.; Puppels, G.J. Identification of medically relevant microorganisms by vibrational spectroscopy. *J. Microbiol. Methods* **2002**, *51*, 255–271.
15. Delcour, J.; Ferain, T.; Deghorain, M.; Palumbo, E.; Hols, P. The biosynthesis and functionality of the cell-wall of lactic acid bacteria. *A. Van Leeuw. J. Microb.* **1999**, *76*, 159–184.
16. Gaus, K.; Rosch, P.; Petry, R.; Peschke, K.D.; Ronneberger, O.; Burkhardt, H.; Baumann, K.; Popp, J. Classification of lactic acid bacteria with UV-resonance Raman spectroscopy. *Biopolymers* **2006**, *82*, 286–290.
17. Mobili, P.; Araujo-Andrade, C.; Londero, A.; Frausto-Reyes, C.; Tzonchev, R.I.; De Antoni, G.L.; Gomez-Zavaglia, A. Development of a method based on chemometric analysis of Raman spectra for the discrimination of heterofermentative *lactobacilli*. *J. Dairy Res.* **2011**, *78*, 233–241.
18. Schär-Zammaretti, P.; Ubbink, J. The Cell Wall of Lactic Acid Bacteria: Surface Constituents and Macromolecular Conformations. *Biophys. J.* **2003**, *85*, 4076–4092.
19. De Gelder, J.; De Gussem, K.; Vandenabeele, P.; Moens, L. Reference database of Raman spectra of biological molecules. *J. Raman Spectrosc.* **2007**, *38*, 1133–1147.
20. Prucek, R.; Ranc, V.; Kvitek, L.; Panacek, A.; Zboril, R.; Kolar, M. Reproducible discrimination between Gram-positive and Gram-negative bacteria using surface enhanced Raman spectroscopy with infrared excitation. *Analyst* **2012**, *137*, 2866–2870.
21. Mobili, P.; Londero, A.; De Antoni, G.; Gomez-Zavaglia, A. Multivariate analysis of Raman spectra applied to microbiology: Discrimination of microorganisms at the species level. *Rev. Mex. Fis.* **2010**, *56*, 378–385.
